# The Journal *Microarrays*

**DOI:** 10.3390/microarrays1010001

**Published:** 2011-10-14

**Authors:** Shu-Kun Lin

**Affiliations:** 1 MDPI AG, Postfach, CH-4005 Basel, Switzerland; Email: lin@mdpi.com

**Keywords:** n/a

## Abstract

Our publishing company MDPI AG has its headquarters in Basel, Switzerland where there are thousands of scientists working in the laboratories of pharmaceutical companies and institutes including Novartis [1], F. Hoffmann-La Roche [2] and institutes affiliated with University of Basel [3]. In 1996, the first annual microplate conference MipTec was held in Basel, and the MipTec 2011 was held a few days ago in Basel [4]. I published a paper on microplate standardization presented at MipTec 1996 in MDPI’s longest-running journal *Molecules* [5-7]. [....]

Our publishing company MDPI AG has its headquarters in Basel, Switzerland where there are thousands of scientists working in the laboratories of pharmaceutical companies and institutes including Novartis [1], F. Hoffmann-La Roche [2] and institutes affiliated with University of Basel [3]. In 1996, the first annual microplate conference MipTec was held in Basel, and the MipTec 2011 was held a few days ago in Basel [4]. I published a paper on microplate standardization presented at MipTec 1996 in MDPI’s longest-running journal *Molecules* [5,6,7].

Due to the rapid development of microarray technologies in recent years, a large number of experiments can be done completely automatically and exhaustively in a combinatorial way. Moreover, today the amount of sample needed―to produce a massive amount of data―is extremely small. For example, Small Molecule Microarray and Micro Arrayed Compound Screening (µARCS) have been developed for screening chemical compound libraries. Incidentally, the precursor of MDPI AG was a nonprofit organization [8] collecting and distributing chemical samples. We are still providing samples services. The very first MDPI journal *Molecules* was launched in 1996 with the promotion of small molecule samples collection as its main purpose.

It is the correct time to have a journal devoted specifically to research activities using microarray technologies. *Microarrays* will be one of MDPI’s open access journals maintaining a speedy but high quality peer review system and fast publishing procedure. A large amount of experimental data can be submitted and deposited as supplementary material. Among the many advantages, open, free access to the main text of the papers and the experimental raw data will greatly facilitate exchange of knowledge among biomedical scientists.

Enjoy publishing with us.
